# Study on quality characteristics of cassava flour and cassava flour short biscuits

**DOI:** 10.1002/fsn3.1334

**Published:** 2019-12-13

**Authors:** Haiqin Lu, Liyun Guo, Lichao Zhang, Caifeng Xie, Wen Li, Bi Gu, Kai Li

**Affiliations:** ^1^ Light Industry and Food Engineering College Guangxi University Nanning China; ^2^ College of Chemistry and Chemical Engineering Guangxi University for Nationalities Nanning China; ^3^ Guangxi Key Laboratory of Chemistry and Engineering of Forest Products Guangxi University for Nationalities Nanning China

**Keywords:** cassava flour, gluten‐free short biscuits, inulin, xanthan gum

## Abstract

In this paper, the basic components, nutrient composition, and processing characteristics of cassava flour were determined. In addition, the effects of xanthan gum and inulin on the pasting properties, microstructure, and thermal properties of cassava flour were studied. Biscuits were prepared using cassava flour as the main raw material and the optimal technology and formula for the biscuits were determined by single‐factor and orthogonal tests. The effects of xanthan gum and inulin on the quality of cassava flour short biscuits were also investigated, and volatile components in the biscuits were determined using electronic nose technique. The addition of xanthan gum improved the pasting properties and microstructure of cassava flour, and improved the taste and increased hardness and brittleness of the biscuits, making their quality similar to that of commercially available short biscuits. The addition of inulin inhibited the setback of starch and improved starch gelatinization. However, inulin was not suitable for processing of cassava flour biscuits as it decreased their hardness, brittleness, and taste. The optimal formula and baking conditions of cassava flour short biscuits were as follows: cassava flour 100 g, water 24 g, shortening 25 g, sugar 30 g, baking powder 0.6 g, salt 1 g, and egg 25 g; the surface fire and primer fire temperatures were 180°C, and the baking time was 9 min. In addition, although the main aroma volatile components present in cassava flour and low gluten wheat flour short biscuits were similar, the proportions of each component were different.

## Graphical abstract

In this study, sweet cassava flour was used as a raw material to prepare gluten‐free (GF) biscuits with addition of xanthan gum and inulin. The effects of the addition of xanthan gum and inulin on the processing characteristics, pasting properties, thermal performance, and microstructure of cassava flour were investigated. Moreover, the effects of xanthan gum and inulin on the quality of cassava flour short biscuits prepared by the best formula were compared with those of commercially available wheat flour short biscuits, to improve the nutritional value and sensory properties of GF biscuits and to provide better GF products for celiac patients.

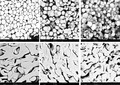

## 1 INTRODUCTION

Cassava is one of the most important starch resources, because it can grow under harsh climatic conditions. Cassava can be cultivated in both tropical and subtropical regions and has become a staple food in those regions, because of its high starch content (Dudu, Lin, Oyedeji, Oyeyinka, & Ying, 2019; Dudu, Oyedeji, Oyeyinka, & Ying, 2019; Kusumayanti, Handayani, & Santosa, 2015). Cassava has been exploited as a raw material for human food production, animal feed, industry, and alternative fuels. In Nigeria, most cassava is processed into various forms for human consumption. Cassava roots have high nutritional value, and they are rich in carbohydrates, which the carbohydrate yield is 40% and 20% higher than in rice and corn, respectively (Bala, Gul, & Riar, 2015). However, the unprocessed cassava root contains cyanogenic glycosides, especially in the fresh and tender parts and cortex. If cassava tissue becomes damaged during harvesting or storage, endogenous enzymes hydrolyze the cyanogenic glycosides and release toxic hydrocyanic acid, which limits the edible value of cassava (Ahaotu et al., 2017). The traditional method of cassava preparation is to remove the skin, chop into pieces, and then remove the cyanogenic glycosides by water immersion or other methods. The pieces are then sun‐dried, ground and cooked, and sometimes combined with other cereals. Indigenous people in South America use cassava powder to make bread, cakes, and other foods, and they also mash and ferment cassava into cassava wine. Indonesia and other Southeast Asian countries use ripe cassava to produce fermented snacks and other special pastries. Among the roots and tubers, cassava is suitable for partial or complete replacement of wheat flour, because of its high yield, low cost of production, and the unique functional properties of its flour and starch (Akingbala, Falade, & Ogunjobi, 2011; Gyedu‐Akoto & Laryea, 2013). Cassava is usually classified as sweet cassava, or bitter cassava based on the content of hydrocyanic acid in the tuberous roots.

Edible grade cassava flour is made from fresh cassava by cleaning, removing inner and outer epidermis, detoxifying (sweet cassava is not used), steaming, slicing, drying, and ultra‐fine grinding. It is rich in a variety of nutrients, including fibers, vitamins, and minerals, and is widely used in the feed, food, and chemical industries (Adesina & Bolaji, 2013). One of the most popular uses of cassava flour is to replace wheat flour for bakery applications (Shittu, Dixon, Awonorin, Sanni, & Maziya‐Dixon, 2008), because of its special quality attributes. Jensen, Skibsted, Kidmose, and Thybo (2015) studied the sensory and textural qualities of bread made by replacing a high gluten flour with cassava flour. Ekunseitan et al. (2017) replaced wheat flour with a high‐quality cassava flour, and mixed wheat flour, mushroom flour, and high‐quality cassava flour in different proportions, to determine the nutritional and functional characteristics of the compound flour. The starch composition, lower retrogradation tendency, melting onset, and water absorption of cassava flour are similar to wheat flour. Nevertheless, cassava flour is deficient in gluten and sulfur‐containing amino acids, and the bakery performance is not good because of its low diastatic activity (Dudu, Lin, et al., 2019; Dudu, Oyedeji, et al., 2019). Consequently, the application of cassava flour in bakery production has technical challenges (Dudu, Lin, et al., 2019; Dudu, Oyedeji, et al., 2019). Cassava flour, however, does not contain gluten and causes no allergic effects when consumed by the patients with celiac disease. Research into gluten‐free (GF) bakery products based on cassava flour would enhance its use in GF products, in particular, when gluten substitutes such as hydrophilic colloids are added to the formulation.

Hydrophilic colloids are widely used as functional ingredients in the food industry for their high molecular weight and hydrophilic characteristics, and they not only improve the taste, texture, and mouthfeel of products, but they also improve the overall quality of the final products to meet production requirements (Kaur, Sandhu, Arora, & Sharma, 2015; Krstonošić, Milanović, & Dokić, 2019). Xanthan gum is a hydrophilic colloid and an acid extracellular heteropolysaccharide, which is widely used in cosmetics, foods, and pharmaceutical industries because of its thickening and emulsifying properties (Bulbul, Bhushette, Zambare, Deshmukh, & Annapure, 2019). Monthe et al. (2019) used xanthan gum as a gluten substitute to produce GF bread with a compound flour made from fermented cassava, sorghum, and sweet potato and evaluated the effects of different combinations of the ingredients on the rheological and textural properties of the dough and bread. Mohammadi, Sadeghnia, Azizi, Neyestani, and Mortazavian (2014) added xanthan gum and carboxymethyl cellulose to the formulation of GF bread. Xanthan gum had more impact on increasing moisture and decreasing hardness. Kaur et al. (2015) found that biscuits prepared from buckwheat flour with gums, compared with those prepared with buckwheat flour alone, demonstrated higher moisture content, weight, diameter, and thickness and decreased fracture strength. The addition of xanthan gum significantly improved the appearance, color, flavor, and overall acceptability of biscuits. Cairano, Galgano, Tolve, Caruso, and Condelli (2018) demonstrated that biscuits, respectively, prepared from foxtail millet flour with xanthan gum and guar gum and quinoa flour with tragacanth and xanthan gum, compared with those prepared from wheat flour, had better sensory and nutritional properties. Xanthan gum seems to be better for improving GF products, compared with other hydrophilic colloids.

Inulin is another hydrophilic colloid and a nonstarch polysaccharide, which is moderately soluble in water, nutritious, and has various applications in the food industry. Inulin has probiotic effects, that is, the ability to improve the intestinal flora, and can be fermented to produce lactic acid and short‐chain fatty acids with participation of the endogenous flora of the colon, which has a positive effect on the digestive system. In food formulations, inulin, as a replacement for sugar or fat, is suitable for consumption by diabetics as it does not affect insulin secretion, or blood glucose levels, and can reduce blood cholesterol levels. Inulin can also improve the bioavailability of calcium, iron, and magnesium by regulating the transit time of food (Bengoechea et al., 2019; Krystyjan, Ciesielski, Khachatryan, Sikora, & Tomasik, 2015; Li, Ma, & Liu, 2019). Moreover, inulin can improve the textural quality of food, because of its good pasting properties. There is currently, strong demand for low‐fat food products with flavor and texture similar to traditional high‐fat products. Addition of 15% inulin to biscuits makes a good fat substitute and gives good sensory qualities (Laguna, Primo‐Martín, Varela, Salvador, & Sanz, 2014). Compared with other common dietary fibers, inulin has better food processing characteristics, so it is frequently added to bakery products, such as bread, biscuits, and steamed bread. Witczak, Witczak, and Ziobro (2014) partially replaced potato starch with pectin and inulin with different degrees of polymerization, and the thermal and rheological properties of starch gels were significantly improved. Hager et al. (2011) added inulin and oat β‐glucan to the formula of GF bread, increasing the specific volume of the bread. Vitali, Dragojević, and Šebečić (2009) added inulin to wheat flour and then mixed with soybean flour, amaranth flour, carob flour, apple fiber, or oat fiber, respectively, and the result indicated that inulin significantly decreased the total energy value of biscuits.

Celiac disease, a chronic enteropathy, is characterized by intolerance to gluten contained in wheat, barley, and other grains, and the only treatment for sufferers is adherence to a strict GF diet throughout their lives. According to EU regulations of 2009, only foods containing less than 20 mg/kg of gluten can be marked with GF on their packaging. Gluten is a protein and plays an important role in the overall sensory and structural characteristics of baked products. It is necessary to replace wheat flour partly or completely with flours that are GF (such as cassava flour, rice flour, and millet flour) to make GF products. Celiac disease is becoming increasingly prevalent internationally, and sufferers tend to have a low dietary fiber intake so there is a need for higher‐fiber GF products (Akingbala et al., 2011; Bala et al., 2015). Biscuits are a relatively cheap, ready‐to‐eat, high‐energy food, popular with all age groups that have a long shelf life (Bala et al., 2015). However, biscuits made from wheat flour are not suitable for celiac disease sufferers (Kurniadi et al., 2019). Gluten plays a relatively minor role in biscuit production compared with that in bread making, so there is considerable research interest in GF biscuits. To enhance the textural qualities of biscuits, gluten substitutes are added, such as xanthan gum, inulin, and pectin. Biscuits made from GF flours can be used as an effective nutritional product for celiac patients (Jan, Panesar, & Singh, 2018a). Jan, Panesar, and Singh (2018b) mixed quinoa flour and wheat flour in different proportions to make biscuits. The biscuits made with quinoa flour had higher nutritional value and crispness, and the fiber and protein contents were increased. There is potential for new GF products, based on changing the formulation and adding functional ingredients.

In this study, sweet cassava flour was used as a raw material to prepare GF biscuits with addition of xanthan gum and inulin. The effects of the addition of xanthan gum and inulin on the processing characteristics, pasting properties, thermal performance, and microstructure of cassava flour were investigated. Moreover, the effects of xanthan gum and inulin on the quality of cassava flour short biscuits prepared by the best formula were compared with those of commercially available wheat flour short biscuits, to improve the nutritional value and sensory properties of GF biscuits and to provide better GF products for celiac patients.

## 2 MATERIALS AND METHODS

### 2.1 Materials

South China No. 9 cassava flour was supplied by Guangxi Science and Technology Board (Wuming). It was prepared by the processes of peeling, shredding (or dicing, slicing, segmenting, etc.), drying, and crushing of the fresh sweet cassava root tubers and was then passed through a 100 mesh screen. Xanthan gum was obtained from Shandong Zhongxuan Co. Ltd., and inulin was purchased from Ruji Biotechnology Development Co. Ltd. Cassava starch and low gluten wheat flour was obtained from Tianzhiyuan Food Co. Ltd. Sugar, salt, and eggs were purchased from a local supermarket. Shortening was procured from Zhirun Oil and Fat Food Industry Co. Ltd. Baking powder and short biscuits were obtained from Angel Yeast Co. Ltd. and Dali Food Group Co. Ltd., respectively.

### 2.2 Basic components and nutrient composition of cassava flour

The basic components (moisture, ash, protein, fat, starch, dietary fiber) and nutrient composition (P, Ca, Mg, Mn, Na, Fe, K, carotene, vitamin C, and various amino acids) of cassava flour were determined according to the national standards of China.

### 2.3 Processing characteristics of cassava flour

#### 2.3.1 Water‐holding capacity

The water‐holding capacity (WHC) of cassava flour was measured following the methods of Du et al. (2018) and Luna and Estévez (2019), with minor modifications. A 2 g sample of cassava flour and 30 ml distilled water were added into a centrifuge tube and stirred well. This tube was then placed in a 40°C water bath, stirred continuously for 20 min, and left to stand for 30 min. Subsequently, the supernatants were discarded by centrifugation at 1,330 *g* for 20 min. The centrifuge tube was weighed after it was drained upside down, and the percentage of mass increase per gram of sample was calculated. Cassava starch was used as a control.

#### 2.3.2 Oil absorption capacity

The oil absorption capacity (OAC) of cassava flour was determined according to the method of Lin, Li, Xu, Jian, and Zhang (2016). A 5 g sample and approximately 30 g peanut oil were added into a centrifuge tube, stirred continuously in a 40°C water bath for 20 min, and left to rest in the water bath for a further 30 min. The tube was then inverted to drain the upper oil slick after it was centrifuged at 1,330 *g* for 20 min, and the percentage of mass increase per gram of sample was calculated. Cassava starch was used as a control.

#### 2.3.3 Freeze–thaw stability

Freeze–thaw stability was evaluated based on the modified method of Babu, Mohan, and Parimalavalli (2019). The sample emulsion with 6% mass fraction was prepared with 6 g cassava flour (dry basis) and was then heated in a boiling water bath for 20 min to allow complete gelatinization, followed by cooling to room temperature. A certain amount of gelatinized cassava flour emulsion was placed in a plastic centrifuge tube of known quality and stored at –18°C–20°C for 24 hr. This tube was then defrosted at room temperature and centrifuged at 1,330 *g* for 20 min, and the quality of the precipitate after leaching was measured. The water evolution rate was calculated using cassava starch as the control, and the formula is as follows:

$$R=\frac{A-B}{A}\times 100\%$$

where *R* is water evolution rate, *A* is the weight of paste, and *B* is the weight of sediment.

### 2.4 Pasting properties

Pasting properties of cassava flour samples were determined using the modified methods of Santos et al. (2018) and Kim, Woo, and Chung (2018). The cassava flour–inulin samples (ratios of cassava flour and inulin were as follows: 10.0:0, 9.5:0.5, 9.0:1.0, 8.5:1.5, and 8.0:2.0) and cassava flour–xanthan gum samples (ratios of cassava flour and xanthan gum were as follows: 10.0:0, 9.5:0.5, 9.0:1.0, 8.5:1.5, and 8.0:2.0) with a total mass of 36.8 g (dry basis) were weighed accurately and placed in a beaker, respectively. Then, a mixture of 8% solid mass fraction was obtained by adding water to a total mass of 460 g, and placing in the measuring cup of a Brabender viscometer (Viscograph‐E, Brabender Corporation) after thorough stirring. The test range was 700 cmg, and the rotation speed was 75 r/min. The samples were heated from 30°C to 95°C at 1.5°C/min, held at 95°C for 30 min, followed by cooling to 50°C at a rate of 1.5°C/min, and maintained at 50°C for 30 min. The Brabender viscosity curves from the corresponding samples were obtained.

### 2.5 Differential scanning calorimetry

Differential scanning calorimetry (DSC) was performed according to Ye et al. (2018), with some modifications. Samples (5 mg) of the cassava flour, cassava starch, cassava flour–inulin (mass ratio was 9.0:1.0), and cassava flour–xanthan gum (mass ratio was 9.0:1.0) were weighed and mixed evenly, respectively, and then placed in an aluminum crucible with 5 µl distilled water. Samples were mixed thoroughly again, and the lid was sealed with a crimp cap. Subsequently, the samples were placed at room temperature for 18 hr and the thermal properties of the samples were analyzed using a DSC (DSC200PC, German Necker Instrument Co. Ltd.). A blank aluminum crucible was used as a reference. The scanning temperature range was 20 to 150°C, and the heating rate was 10°C/min. The results were analyzed using the instrument software.

### 2.6 Microstructure characteristics

The microstructure characteristics of the samples were evaluated according to Martins, Gutkoski, and Martins (2018). Cassava flour, cassava starch, cassava flour–inulin (mass ratio was 9.0:1.0), and cassava flour–xanthan gum (mass ratio was 9.0:1.0) suspensions with a 6% mass fraction (dry basis) were prepared and then hydrated for 20 min on a magnetic stirrer (HJ‐6A, Changzhou Putian Instrument Manufacturing Co. Ltd.). A portion of the samples was gelatinized in a hot water bath at 95°C for 15 min and then freeze‐dried for 48 hr. The microstructure was observed using a scanning electron microscope (SEM; S‐3400N‐II, Hitachi High‐Tech/EDAX Inc.), operating at 2,000 × magnification with an accelerating voltage of 10 kV after spraying gold with ion sputtering apparatus.

### 2.7 Analysis of biscuits

#### 2.7.1 Determination of optimal formulation and baking conditions

On the basis of previous experiments, the basic formula for biscuit formation was as follows: cassava flour 100 g, sugar 30 g, salt 1 g, shortening 20 g, water 18 g, baking powder 0.8 g, and egg 25 g. Referring to the basic formula and on the basis of single‐factor analysis, the L_9_(3^4^) orthogonal test was carried out by selecting the amount of water, shortening, sugar, and baking powder as the experimental factors and setting different levels for each factor. In addition, on the basis of previous work, the L_4_(2^3^) orthogonal test was carried out by selecting three factors, that is, the surface fire, the primer fire, and the baking time. Finally, the optimal formula and the optimal baking conditions were determined. The orthogonal test designs are shown in Tables 1 and 2, respectively.

**Table 1:** Orthogonal test design showing contents of water, shortening, sugar, and baking powder used in the experiment

Factors	Levels		
A. Water addition/g	18	21	24
B. Shortening addition/g	20	25	30
C. Sugar addition/g	25	30	35
D. Baking powder addition/g	0.6	0.8	1.0

**Table 2:** Orthogonal test design showing levels of surface fire, primer fire, and baking time used in the experiment

Factors	Levels	
A. Surface fire/°C	180	195
B. Primer fire/°C	180	195
C. Baking time/min	9	11

#### 2.7.2 Texture and sensory evaluation of biscuits

The texture quality of biscuits was measured using a texture analyzer (TA.XT.plus, Stable Micro Systems Inc.). The hardness and brittleness of the biscuits were determined by puncture and compression modes, respectively.

1. The hardness of biscuits was measured by the puncture test with a P5 cylindrical probe. The specific test parameters were as follows: test mode, compression; pretest speed, 2 mm/s; test speed, 3 mm/s; post‐test speed, 10 mm/s; straining, 75%; trigger force, 5 g; trigger mode, automatism; and data acquisition rate, 200 pps.
2. The brittleness of biscuits was determined by using HDP/CFS, that is, a P/0.25S spherical probe. The test conditions were as follows: test mode, puncture; pretest speed, 1 mm/s; test speed, 1 mm/s; post‐test speed, 10 mm/s; distance, 3 mm; trigger force, 5 g; trigger mode, automatism; and data acquisition rate, 500 pps.

Ten parallel samples were taken for each group, and the average values of the measured data were recorded.

Referring to the improved evaluation criteria based on the biscuit standard GB/T 20980‐2007, the sensory evaluation of the products (i.e., shape, color, taste, mouthfeel, and texture) was examined. The sensory evaluation criteria are shown in Table 3.

**Table 3:** Sensory evaluation criteria of cassava flour biscuits

Quality parameter	Index	Score
Shape	Complete shape, uniform thickness, no deformation, no bubbles, no cracks, no larger or more concave bottom	30
Color	Uniform color, no over‐coke and over‐white phenomena	20
Taste and mouthfeel	Cassava flavor, no odor, crisp taste, not dry, nonstick teeth	30
Texture	The section structure is porous, fine and no big holes	20

#### 2.7.3 Effects of xanthan gum and inulin on texture and sensory properties of biscuits

According to the results of the single‐factor and orthogonal tests, the optimum formula of cassava flour biscuits was obtained. The usage of xanthan gum in food refers to standards for the use of food additives in the national standard for food safety (GB 2760‐2014); that is, the maximum usage of xanthan gum in food was 10.0 g/kg. On the basis of the optimal formula, 1% xanthan gum and inulin were added, respectively, to make the short biscuits, and the effects of xanthan gum and inulin on the sensory and textural quality of the biscuits were observed and contrasted with the short biscuits sold commercially.

### 2.8 Volatile aroma

Referring to the optimal formula and baking conditions obtained from Section 2.7.1 of this paper, short biscuits were made using low gluten wheat flour and cassava flour as the main raw materials, respectively.

#### 2.8.1 Electronic nose detection

The biscuit samples made from low gluten wheat flour and cassava flour were weighed into 20 g, then crushed, and sealed in a 50‐ml small beaker. Thereafter, samples were heated in a 45°C water bath for 30 min and detected by manual headspace injection with an electronic nose (PEN3, AIRSENSE Inc.).

Test conditions were as follows: Carrier gas was clean and dry air; sampling time interval, 1 s; cleaning time, 100 s; zero adjustment time, 10 s; presampling time, 5 s; measuring time, 100 s; sensor chamber flow rate, 400 ml/min; measuring sample flow rate, 400 ml/min; maximum G/G0, 3.0; and sensor, 8.

### 2.9 Data analysis

The data obtained by DSC were analyzed using the instrument's own software. The rest of the data were analyzed by Origin 8.0 software and tested at a significance level of *p* < .05.

## 3 RESULTS AND DISCUSSION

### 3.1 Basic chemical components and nutrient compositions

The fat content of cassava flour was lower, and the ash and starch contents were higher than those of low gluten wheat flour. The protein content of cassava flour was substantially lower than wheat flour (Table 4). Starch and protein represent a high proportion of wheat flour and cassava flour, and they are also the main components which determine processing characteristics and product quality. The processing characteristics of flour, such as WHC, OAC, and freeze–thaw stability, are affected by the content of starch. Protein plays a unique functional role in food, and its content and quality can have a major influence on the structure and quality of the product. Cassava flour does not contain gluten protein, and therefore, it can be used to develop GF foods. However, the protein content in cassava flour is relatively low, which limits its processing characteristics and end product quality.

**Table 4:** Basic chemical components (%) of cassava flour and low gluten wheat flour

Samples	Moisture	Ash	Protein	Fat	Starch	Dietary fiber (DF)
Cassava flour	11.9	2.0	2.8	0.6	76.4	2.0
Low gluten wheat flour	12.6	0.3	8.5	1.2	71.23	1.9

Sweet cassava flour is nutritious and well‐balanced (Tables 5 and 6). It is rich in dietary fiber, vitamin C, amino acids, and other nutrients. There were many types of amino acid present, including essential amino acids, such as val, lle, leu, and lys, and the flour also contained minerals such as P, Ca, Mg, Fe, and K. The contents of the various nutrients in cassava flour were significantly higher than those in cassava starch, and the types of components were more complete. Overall, cassava flour had a higher nutritional value than cassava starch. Since cassava flour does not contain gluten, it can be used as a GF raw material in biscuit production to improve the nutritional value of biscuits.

**Table 5:** Nutrient composition of sweet cassava flour and cassava starch

Inspection items	Units	Sweet cassava flour (noninstant food, GR891)	Cassava starch (food grade)
Hydrocyanic acid	mg/kg	1.6	0.077
P	mg/100 g	18.43	9.58
Ca	mg/100 g	2,708	61.5
Mg	mg/100 g	163	2.39
Mn	mg/100 g	5.90	0.24
Na	mg/100 g	31.1	0.99
Fe	mg/100 g	37.0	1.60
K	mg/100 g	401	20.6
Dietary fiber (DF)	g/100 g	4.82	0.34
Total amino acids	g/100 g	671.7	0.03
Carotene	mg/kg	0.92	Not detected
Protein	g/100 g	1.84	0.50
Vitamin C	mg/100 g	2.08	1.59

**Table 6:** Amino acid compositions of cassava flour in Wuming station

Amino acids	(mg/100 g)
Asp	0.13
Thr	0.03
Ser	0.03
Glu	0.29
Pro	0.03
Gly	0.03
Ala	0.03
Cys	0.00
Val	0.03
Met	0.02
lle	0.03
Leu	0.04
Tyr	0.02
Phs	0.03
Lys	0.06
NH_3_	0.04
His	0.03
Arg	0.63
Total amino acids	1.46

### 3.2 WHC, OAC, and freeze–thaw stability

The WHC and OAC characteristics of the raw material flour have significance in guiding food development. In the processing process, good WHC can increase the amount of water appropriately. Good OAC can appropriately increase the amount of oil, and the amount of oil has a great effect on the plasticity and appearance of the dough, especially in baking products. The WHC of cassava flour was better than that of cassava starch (Table 7). This may be because the processing of cassava flour changed the structure of the starch granules, which increased the specific surface area and consequently the WHC. The OAC of cassava flour was also slightly better than that of cassava starch, which may be related to the protein content. During gelatinization, the spatial structure of protein is stretched and opened, and some hydrophobic groups are exposed. Therefore, cassava flour is more suitable for the processing of baked goods. The freeze–thaw stability can reflect the stability of samples during cold processing or storage, and the evaluation index is the water evolution rate. The lower the water evolution rate, the better the stability at low temperature. The water evolution rate of cassava flour was lower than that of cassava starch, meaning that cassava flour had better freeze–thaw stability and can be used for the manufacture of frozen foods.

**Table 7:** Water‐holding capacity, oil absorption capacity, and water evolution rate of cassava flour and cassava starch used in the current study

Samples	WHC/(g/g)	OAC/(g/g)	Water evolution rate/%
Cassava flour	1.23	0.85	1.51
Cassava starch	0.68	0.70	1.92

### 3.3 Pasting properties

Table 8 shows the effects of addition of different amounts of xanthan gum on the pasting properties of cassava flour. The addition of xanthan gum reduced the onset pasting temperature of cassava flour. When the mass ratio of cassava flour to xanthan gum was 8.5:1.5 and 8.0:2.0, the onset pasting temperature of the system increased. With the addition of increasing amounts of xanthan gum, the peak viscosity and final viscosity of the system significantly (*p* < .05) increased. The results showed that the xanthan gum–cassava flour complex system would be a more suitable thickener in foods than cassava flour alone. The breakdown of the system first increased and then decreased. When the amount added was less than 10%, the shear thinning behavior of the system was more obvious with an increase of xanthan gum; when 10%–20% was added, the shear resistance of the system was enhanced when xanthan gum was increased. This may be because when the amount of xanthan gum added is less, it reacts with starch through hydrogen bonds in the process of heating gelatinization. Then, the starch granules absorb water and expand. When the concentration of xanthan gum is increased further and the viscosity of the system is improved, it results in a larger shear force acting on starch granules and makes them more easily deformed and damaged; that is, the breakdown increases. With the addition of increased amounts of xanthan gum, the trisaccharide side chain and main chain are arranged tightly in a straight line to form a clava with elasticity and stability, which can form a firm three‐dimensional network structure with the starch molecular chain segments of cassava flour, thus reducing the breakdown. When the levels of xanthan gum were increased, the setback decreased at first, then increased, and finally decreased again. This may be due to the fact that xanthan gum can prevent the formation of hydrogen bonds between starches and reduce the setback, but the effects of hydrogen bonds approaching each other between xanthan gum and amylose also increased the setback. The setback decreased again which may be due to the formation of a three‐dimensional network structure between a clava formed by xanthan gum and starch molecules.

**Table 8:** Parameters of pasting characteristics of cassava flour and cassava flour–xanthan gum blends

Cassava flour/xanthan gum, ratio (g/g)	Pasting‐onset temperature/°C	Peak viscosity/BU	Breakdown/BU	Setback/BU	Final viscosity/BU
10.0:0	59	698	424	138	375
9.5:0.5	36.8	826	453	114	470
9.0:1.0	36.3	1,260	492	179	959
8.5:1.5	54.1	1,198	449	151	917
8.0:2.0	53.3	1,328	373	118	1,097

The addition of different proportions of inulin increased the pasting temperature of cassava flour (Table 9). This may be due to the strong hygroscopicity of inulin, which may have been competing with starch in cassava flour for water needed for gelatinization, which subsequently prevented the cassava flour from absorbing water and swelling and making it more difficult to gelatinize. With the addition of greater amounts of inulin, the peak viscosity and final viscosity decreased significantly (*p* < .05). Inulin promoted the binding of amylose to amylopectin, and therefore, amylopectin could not fully combine with water; that is, hydration of the starch granules decreased, and therefore, the peak viscosity of the system decreased. In addition, inulin is distributed around the starch granule and encases the starch granules, which limits the release of amylose. Alternatively, inulin may have interfered with the interaction between amylose particles and therefore reduced the viscosity of the system. Thus, it could be assumed that inulin can inhibit in vitro digestion of starch by amylase and thus can inhibit the digestion of starch and delay the blood glucose response. Therefore, inulin could be used for the development of hypoglycemic index products. When inulin levels were increased, the breakdown of the system decreased, indicating that inulin reduced the damaging effects of shear force on starch granules. The setback of starch is affected by the fine structure of amylopectin, the ratio of amylose to amylopectin and the moisture content (Salinas & Puppo, 2013). After addition of inulin, the setback of the system gradually decreased, indicating that the addition of inulin significantly inhibited the setback of starch. Inulin may be able to prevent or reduce the recrystallization of amylose after gelatinization, which would improve the stability of cassava flour foods during storage.

**Table 9:** Parameters of pasting characteristics of cassava flour and cassava flour–inulin blends

Cassava flour/xanthan gum, ratio (g/g)	Pasting‐onset temperature/°C	Peak viscosity/BU	Breakdown/BU	Setback/BU	Final viscosity/BU
10.0:0	59	698	424	138	375
9.5:0.5	65.6	633	372	128	350
9.0:1.0	66.2	526	272	101	341
8.5:1.5	66.7	464	213	77	302
8.0:2.0	67.1	422	181	64	279

### 3.4 DSC analysis

**Figure 1:**
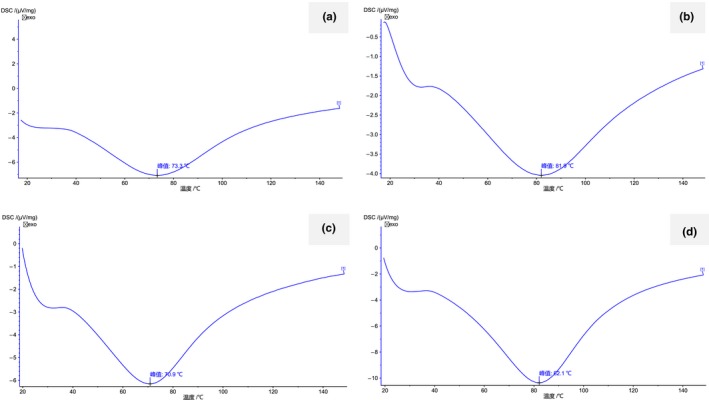
DSC curves from (a) cassava starch, (b) cassava flour, (c) cassava flour–xanthan gum, and (d) cassava flour–inulin

The DSC curves of cassava flour, cassava starch, cassava flour–xanthan gum, and cassava flour–inulin are shown in Figure 1. The pasting parameters obtained are shown in Table 10. The Ton, Tp, and Toff of gelatinization of cassava flour were higher than those of cassava starch, whereas the enthalpy was lower, indicating that the thermal stability of cassava flour was better than that of cassava starch. Compared with cassava starch, the starch granules in cassava flour were not separated out, and most of them were in intact cells; therefore, gelatinization was delayed slightly with an increase in temperature. The enthalpy of cassava flour was lower than that of cassava starch, suggesting that less energy was needed to destroy the starch structure in cassava flour. After adding xanthan gum, the Ton, Tp, and Toff of gelatinization decreased, whereas these parameters were increased by the addition of inulin, which was consistent with the results in Section 3.3. Furthermore, the addition of xanthan gum and inulin increased the enthalpy of the system. This may be explained by xanthan gum and inulin dissolving in water to form a gel and reducing the free water in the system. This resulted in a tight starch chain structure and a stronger connection between starch granules, requiring more energy to break down the structure.

**Table 10:** Gelatinization peak thermal parameters, of cassava starch, cassava flour, cassava flour–xanthan gum, and cassava flour–inulin, measured by differential scanning calorimetry (DSC)

Samples	Onset temperature (Ton)/°C	Peak temperature (Tp)/°C	Offset temperature (Toff)/°C	Enthalpy/(J/g)
Cassava starch	39.7	73.3	109.7	1.09
Cassava flour	42.8	81.9	112.4	0.62
Cassava flour–xanthan gum	40.9	70.9	98.8	0.90
Cassava flour–inulin	46.2	82.1	107.8	1.70

### 3.5 Microstructure characteristics

**Figure 2:**
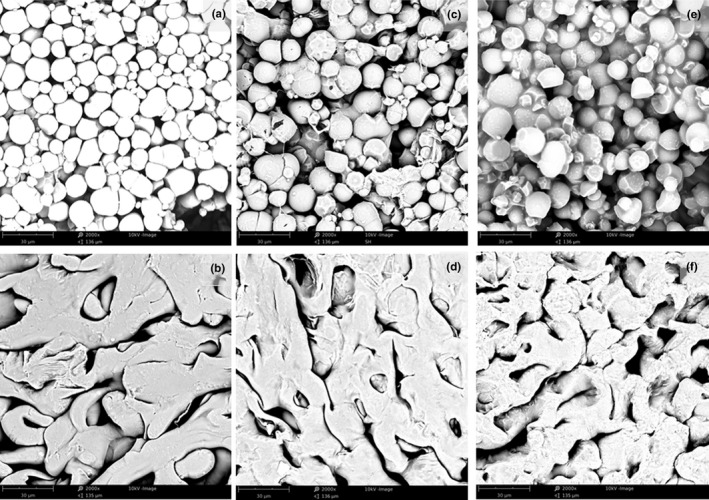
Scanning electron micrograph of (a) cassava flour (6%), (b) cassava flour (6%) treated at 95°C, (c) cassava flour/xanthan gum (mass ratio 9.0:1.0), (d) cassava flour/xanthan gum (mass ratio 9.0:1.0) treated at 95°C, (e) cassava flour/inulin (mass ratio 9.0:1.0), and (f) cassava flour/inulin (mass ratio 9.0:1.0) treated at 95°C

Cassava flour (Figure 2a) shows a large number of intact starch granules, which were prominent and distributed uniformly. However, there was only simple stacking and also gaps between the granules. At this time, the temperature did not reach the pasting‐onset temperature and the granules did not absorb water and swell. Therefore, the integrity of the granules was maintained and the viscosity of the system was relatively low. The cassava starch granules in Figure 2b had absorbed water and were swollen, although some were not gelatinized completely, the number of intact starch granules was decreased, and there were a lot of starch granule fragments in the system. During heating, the starch granules absorbed water, swelled, and then ruptured. The connection between the granules was obviously enhanced, and the viscosity of the system was higher than that observed in Figure 2a. However, there were also many holes visible and the distribution was not uniform.

Figure 2c,e shows the microstructural characteristics of the cassava flour–xanthan gum and cassava flour–inulin suspensions, respectively. Xanthan gum and inulin addition both filled the gaps between the starch granules. The viscosity of the system increased, and the starch granules were no longer uniformly dispersed. Xanthan gum and inulin form viscous gels in water, which makes starch granules adhere to each other. However, the adherence between granules was not uniform, and there were still large gaps visible.

Figure 2d,f shows the microstructural characteristics of cassava flour–xanthan gum and cassava flour–inulin suspensions, respectively, after heating at 95°C. Most starch granules had dissolved and ruptured to form a high viscosity matrix, and fewer intact granules were visible; gelatinization was more extensive with xanthan gum and inulin present. Fewer gaps were visible, the structure was more compact, and the distribution was more uniform.

Clearly, the addition of xanthan gum and inulin made the texture of the system more uniform and improved the pasting properties of cassava flour.

### 3.6 Biscuit analysis

#### 3.6.1 Determination of optimal formulation

The effects of different amounts of water, shortening, sugar, and baking powder on the brittleness, hardness, and sensory quality of cassava flour biscuits were studied by single‐factor tests (see Appendix S1), and the optimal level of each factor was determined (Section 2.7.1). The orthogonal test results of formula optimization are shown in Table 11. The order of importance of the variables on the sensory quality of cassava flour biscuits was as follows: sugar addition (C) > baking powder addition (D) > shortening addition (B) > water addition (A), and the optimal formula was A_3_B_2_C_1_D_2_. This formula was not one of the orthogonal test combinations, and it was important to confirm its sensory and textural qualities. The sensory score was 87, the hardness was 2,535.892 g, and the brittleness was 1,089.724 g. This confirmed that the optimal ratio of each factor was A_3_B_2_C_1_D_2_; that is, the optimal formula was 30 g sugar, 0.6 g baking powder, 25 g shortening, and 24 g water.

**Table 11:** Orthogonal test results of biscuit formula optimization based on sensory score

Test number	Factors				Sensory score
	(A) Water addition/g	(B) Shortening addition/g	(C) Sugar addition/g	(D) Baking powder addition/g	
1	1	1	1	1	75
2	1	2	2	2	84
3	1	3	3	3	60
4	2	1	2	3	74
5	2	2	3	1	70
6	2	3	1	2	77
7	3	1	3	2	67
8	3	2	1	3	69
9	3	3	2	1	72
*k*_1_	73.000	72.000	73.667	72.333	
*k*_2_	73.667	74.333	76.667	76.000	
*k*_3_	69.333	69.667	65.667	67.667	
Range	4.333	4.667	11.000	8.333	
Deviation squared sum	32.667	32.667	194.000	104.667	
*F* ratio	0.359	0.359	2.132	1.150	
*F*_0.05_ critical value	4.460	4.460	4.460	4.460	

The orthogonal test results of the baking conditions are shown in Table 12. The order of importance of the variables on the sensory qualities of cassava flour biscuits was as follows: primer fire (B) > surface fire (A) > baking time (C), and the optimal baking conditions were A_2_B_2_C_1_; that is, the surface fire and primer fire were 180°C and the baking time was 9 min. These conditions resulted in evenly colored biscuits, with no coke on the surface or bottom, no incomplete cooking, a crisp taste, and the highest quality.

**Table 12:** Orthogonal test results of baking temperature and time based on sensory score

Test number	Factors			Sensory score
	A Surface fire/°C	B Primer fire/°C	C Baking time/min	
1	1	1	1	59
2	1	2	2	72
3	2	1	2	67
4	2	2	1	82
*k*_1_	65.500	63.000	70.500	
*k*_2_	74.500	77.000	69.500	
Range	9	14	1	
Deviation squared sum	81	196	1	
*F* ratio	0.874	2.115	0.011	
*F*_0.05_ critical value	10.100	10.100	10.100	

#### 3.6.2 Effects of xanthan gum and inulin on biscuits

The effects of xanthan gum and inulin on the textural qualities of cassava flour short biscuits are shown in Table 13. The addition of 1% xanthan gum increased the hardness and brittleness of cassava flour biscuits and made their textural quality close to that of commercial biscuits. These results are consistent with the effects of xanthan gum on the overall sensory quality of cassava flour biscuits. The addition of xanthan gum resulted in biscuits which had improved taste and were not dry. Furthermore, the hardness of the biscuits had increased, they were not fragile, the cross‐section was more ordered, and the acceptability was better. However, the addition of inulin had the opposite effect on the quality of biscuits. These biscuits had reduced hardness and brittleness, and inulin did not improve their slightly dry taste. The hardness and brittleness of biscuits is required for storage and transportation purposes, to ensure that the biscuits are not fragile, and to maintain the integrity of shape and better taste. Therefore, inulin was not suitable for the processing of cassava flour biscuits, while xanthan gum improved the quality of cassava flour short biscuits and resulted in quality similar to that of commercially available wheat flour short biscuits.

**Table 13:** Comparisons of hardness and brittleness of biscuits made with the optimal cassava flour formula, optimal formula plus 1% xanthan gum, or 1% inulin and commercial wheat flour short biscuits

Biscuit formulas	Optimal formula	Optimal formula plus 1% xanthan gum	Optimal formulation plus 1% inulin	Wheat flour short biscuit on sale
Hardness/g	2,535.892	3,537.581	2,019.720	3,545.994
Brittleness/g	1,089.724	2,323.637	905.560	2,109.766

### 3.7 Volatile aroma components

#### 3.7.1 Electronic nose detection

**Figure 3:**
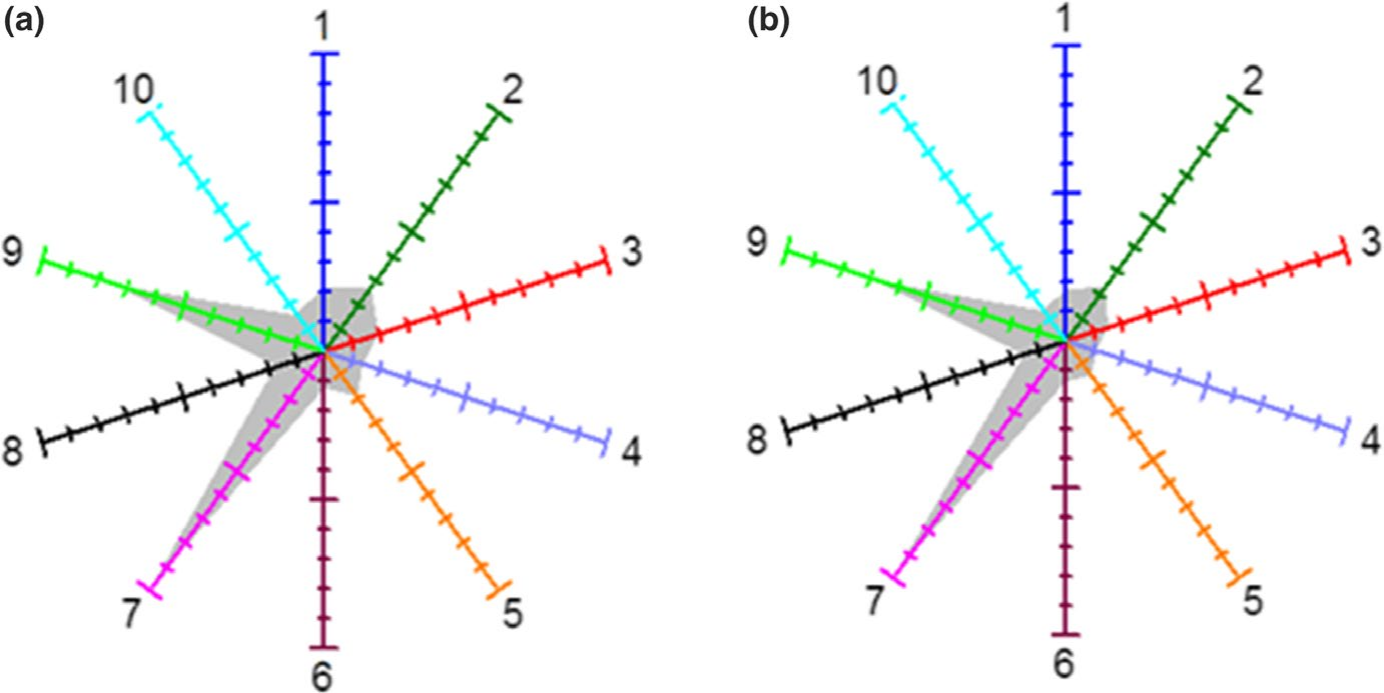
Radar chart of volatile components in (a) low gluten wheat flour biscuits and (b) cassava flour short biscuits

Electronic nose detection and analysis of volatile components in low gluten wheat flour and cassava flour short biscuits was performed. The intensity curves of the electronic nose sensor can be used to characterize the intensity of each volatile component in the sample. The variation trend of the signal values of the 10 sensors was consistent with the intensity difference of the volatile components (Figure S9). The radar chart was established based on the corresponding values between the sensors connected to each other (Figure 3). The flavor intensity profiles of the low gluten wheat biscuits and the cassava flour samples were roughly the same; that is, they contained similar volatile components. The flavor components were mainly aromatics, nitrogen oxides, sulfur compounds, ammonia, and alkanes.

The two types of biscuits could be clearly separated, which indicated that the volatile components and their contents in the samples were different, and the electronic nose quickly distinguished between the two types of biscuits by measuring the concentration of each volatile component in the samples (Figure S10). The contribution rate of the first principal component was 98.671%, the second principal component was 1.2065%, and the total contribution rate was 99.878%. Therefore, it can be concluded that these two principal components can better reflect the difference in flavor components between low gluten wheat flour and cassava flour short biscuits.

## 4 CONCLUSIONS

Addition of xanthan gum and inulin to cassava flour significantly modified the pasting properties, thermal properties, and microstructure. Short biscuits were prepared with cassava flour as the main ingredient, and the optimal formula and baking conditions were determined, as well as the effects of xanthan gum and inulin on biscuit quality. Addition of xanthan gum improved both cassava flour properties and biscuit quality. Though inulin could inhibit the setback of starch and improve starch gelatinization, it decreased the hardness, brittleness, and taste of biscuits. The cassava flour short biscuits made by the optimal cassava flour formula were acceptable, and their main volatile components were similar to those of low gluten wheat flour short biscuits. We conclude that it is feasible to make GF biscuits with cassava flour and the biscuit quality can be improved by adding xanthan gum as a gluten substitute. The literature on celiac disease, discussed above, indicates that these new biscuits should be very suitable for celiac sufferers to eat as part of their GF diet; however, our future work will aim to verify that this is correct.

## CONFLICTS OF INTEREST

There is no conflict of interest.

## ETHICAL STATEMENT

This study does not involve any human or animal testing.
