# Supplementary material for: Study on quality characteristics of cassava flour and cassava flour short biscuits
Source: Food Sci Nutr. 2019 Dec 13;8(1):521–33. doi: 10.1002/fsn3.1334 (PMC6977506; doi:10.1002/fsn3.1334)
Supplement: Supplementary file 1 [file FSN3-8-521-s001.doc]

**Supplementary material**

**Study on quality characteristics of cassava flour and cassava flour short biscuits**

**Haiqin Lu ^a^, Liyun Guo ^a^,** **Lichao Zhang^a^, Caifeng Xie ^a^,** ***, Wen Li ^b,c^,** ***,** **Bi Gu ^a^, Kai Li ^a^**

**^a^** Light Industry and Food Engineering College, Guangxi University, Nanning, 530004, China;

**^b^** College of Chemistry and Chemical Engineering, Guangxi University for Nationalities, Nanning, 530008, China;

**^c^** Guangxi Key Laboratory of Chemistry and Engineering of Forest Products, Guangxi University for Nationalities, Nanning, 530008, China;

* Corresponding authors.

E-mail addresses: [liwen@gxun.edu.cn](mailto:liwen@gxun.edu.cn) (Wen Li); [fcx11@163.com](mailto:fcx11@163.com) (Caifeng Xie).

**Biscuit preparation**

**Process used**

Premix of raw and auxiliary materials → roll press (over press to ensure consistency of thickness, about 3 mm) → molding → baking → cooling → finished product.

(1) Premix of raw and auxiliary materials

Sugar and salt were dissolved in water, baking powder, eggs and shortening were added, mixed evenly, finally, cassava flour was added and mixed well to form the dough.

(2) Roll press

Dough was put on a fresh-keeping bag, rolled to about 5 mm thickness, covered with another fresh-keeping bag, pressed with a pressing machine, adjusted to 3 mm roller spacing, 3–5 times, to achieve a uniform thickness.

(3) Molding

The fresh-keeping bag was removed. The biscuit billet was obtained by manual pressing (uniform force) with an impression mold and placed on a baking tray covered with oiled paper.

(4) Baking

The surface fire and primer fire temperatures of the oven were 160°C and 195°C, preheated for 3 minutes, then the baking tray was placed in the middle of the oven. The biscuit billets were baked until ripe, removed from the oven, cooled to room temperature, then sealed and preserved.

**Single factor test of biscuit formula**

According to the basic formula, the addition of water, shortening, baking powder and sugar were changed, and the water additions were 15 g, 18 g, 21 g, 24 g, 27 g; the shortening additions were 10 g, 15 g, 20 g, 25 g, 30 g; the sugar additions were 20 g, 25 g, 30 g, 35 g, 40 g; the baking powder additions were 0.4 g, 0.6 g, 0.8 g, 1.0 g, 1.2 g, respectively.

(1) Effect of water addition on hardness, brittleness and sensory quality of cassava flour biscuits

**Fig. S1.** Effect of water addition on hardness and brittleness of cassava flour biscuits.

**Fig. S2.** Effect of water addition on sensory quality of cassava flour biscuits.

The effects of water addition on the hardness, brittleness and sensory quality of cassava flour biscuits are shown in Supplementary Fig. S1 and Supplementary Fig. S2, respectively. The hardness and brittleness of biscuits increased with increased water addition (Supplementary Fig. S1). When the water addition was 15 g, the starch and protein in cassava flour could not absorb water fully and could not form dough. The biscuits obtained were loose and dry in taste, not crisp. When the water addition was 27 g, the dough was wet and sticky, and the roll molding was difficult. The sensory score of biscuits increased at first and then decreased with the increase of water addition (Supplementary Fig. S2). Sensory quality was best at a water addition of 21 g, the shape was complete without cracks, there were no big bubbles, and the texture was crisp and non-sticky. Therefore, the optimal water addition was determined to be 21 g.

(2) Effect of shortening addition on hardness, brittleness and sensory quality of cassava flour biscuits

**Fig. S3.** Effect of shortening addition on hardness and brittleness of cassava flour biscuits.

**Fig. S4.** Effect of shortening addition on sensory quality of cassava flour biscuits.

The effects of shortening addition on the hardness, brittleness and sensory quality of cassava flour biscuits are shown in Supplementary Fig. S3 and Supplementary Fig. S4, respectively. With increased shortening addition, the hardness of biscuits decreased gradually and the brittleness increased at first, then decreased (Supplementary Fig. S3). At a shortening addition of 10 g, the dough was hard, the biscuit surface was cracked and rough, some biscuits had concave undersides, the hardness was high and the sensory quality was poor. When the shortening addition was 30 g, the biscuits were too crisp and fragmented and the mouthfeel was too greasy. At a shortening addition of 25 g, the biscuits had complete shape, no fragmentation, crisp taste, good sensory quality and acceptability. The optimum shortening addition was determined to be 25 g.

(3) Effect of sugar addition on hardness, brittleness and sensory quality of cassava flour biscuits

**Fig. S5.** Effect of sugar addition on hardness and brittleness of cassava flour biscuits.

**Fig. S6.** Effect of sugar addition on sensory quality of cassava flour biscuits.

The effects of sugar addition on the hardness, brittleness and sensory quality of cassava flour biscuits are shown in Supplementary Fig. S5 and Supplementary Fig. S6, respectively. The hardness and brittleness of biscuits increased gradually with increased sugar addition (Supplementary Fig. S5). When the sugar addition was about 20 g, the dough was slightly hard, the biscuits were lighter in color, dry in texture and some of them had cracks on the underside. When the sugar addition reached 40 g, the biscuits were uniformly colored and flat, but the taste was too sweet and the acceptability was slightly worse. Thus, the optimal sugar addition was 30 g.

(4) Effect of baking powder addition on hardness, brittleness and sensory quality of cassava flour biscuits

**Fig. S7.** Effect of baking powder addition on hardness and brittleness of cassava flour biscuits.

**Fig. S8.** Effect of baking powder addition on sensory quality of cassava flour biscuits.

The effects of baking powder addition on the hardness, brittleness and sensory quality of cassava flour biscuits are shown in Supplementary Fig. S7 and Supplementary Fig. S8, respectively. With increased addition of baking powder, the hardness of biscuits decreased and the brittleness increased gradually (Supplementary Fig. S7). At an addition of 0.4 g, the taste of biscuits was hard and some biscuits were cracked. At an addition of 1.2 g, some biscuits had bubbles on the surface or large holes in the interior, and the structure was not uniform. Thus, the optimal baking powder addition was 0.8 g.

**Electronic nose detection**


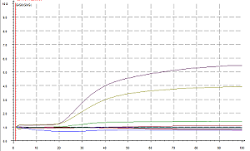

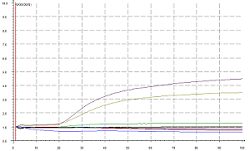


**(a)**

**(b)**

**Fig. S9.** Intensity curves of electronic nose sensor for volatile components of (a) low gluten wheat flour biscuits and (b) cassava flour short biscuits.


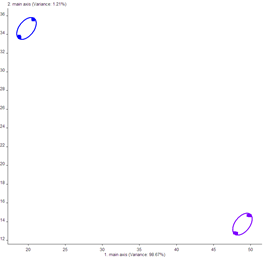

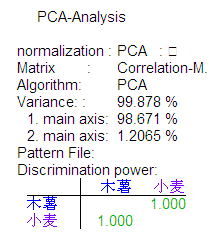


**Fig. S10.** Principal component analysis diagram of volatile components in low gluten wheat flour and cassava flour short biscuits.
